# Pain Management Program in Cardiology: A Template for Application of Normalization Process Theory and Social Marketing to Implement a Change in Practice Quality Improvement

**DOI:** 10.3390/ijerph19095251

**Published:** 2022-04-26

**Authors:** Kerstin Bode, Peter Whittaker, Miriam Dressler, Yvonne Bauer, Haider Ali

**Affiliations:** 1 Department of Electrophysiology, Heart Center Leipzig, Struempellstr. 39, 04289 Leipzig, Germany; y.bauer78@gmail.com; 2 Department of Cardiology, Asklepios Clinic Weißenfels, Naumburger Str. 76, 06667 Weissenfels, Germany; 3 The University of Edinburgh, Old College, South Bridge, Edinburgh EH8 9YL, UK; p.whittaker@sms.ed.ac.uk; 4 Medical Faculty, University of Leipzig, Liebigstr. 21, 04109 Leipzig, Germany; miriam.doerschner@gmx.de; 5 Business School, The Open University, Walton Hall, Milton Keynes MK7 6AA, UK; haiderali@mac.com

**Keywords:** cardiac ablation, cardiac electrophysiology, co-creation, EPOC, normalization process theory, pain management program, pacemaker, peri-operative management, tangibility

## Abstract

Quality improvement plays a major role in healthcare, and numerous approaches have been developed to implement changes. However, the reasons for success or failure of the methods applied often remains obscure. Normalization process theory, recently developed in sociology, provides a flexible framework upon which to construct quality improvement. We sought to determine if examination of a successful quality improvement project, using normalization process theory and social marketing, provided insight into implementation. We performed a retrospective analysis of the steps taken to implement a pain management program in an electrophysiology clinic. We mapped these steps, and the corresponding social marketing tools used, to elements of normalization process theory. The combination of mapping implementation steps and marketing approaches to the theory provided insight into the quality-improvement process. Specifically, examination of the steps in the context of normalization process theory highlighted barriers to implementation at individual, group, and organizational levels. Importantly, the mapping also highlighted how facilitators were able to overcome the barriers with marketing techniques. Furthermore, integration with social marketing revealed how promotion of tangibility of benefits aided communication and how process co-creation between stakeholders enhanced value. Our implementation of a pain-management program was successful in a challenging environment composed of several stakeholder groups with entrenched initial positions. Therefore, we propose that the behavior change elements of normalization process theory combined with social marketing provide a flexible framework to initiate quality improvement.

## 1. Introduction

Pain is disabling and often adds to costs [1]. Consequently, pain management has attracted attention [2,3]. One focus has been on assessment of pain-management programs [4]. For example, examination of pain-management guidelines for healthcare workers [5] and communication with patients about pain management after cardiac surgery [6].

Many procedures in cardiac electrophysiology are minimally invasive, and therefore post-interventional pain management programs appear unnecessary [7,8]. Nevertheless, even minor, minimally invasive interventions, such as percutaneous cardiac ablation for atrial fibrillation, followed by bed rest can result in back pain. Similarly, implantation of pacemakers or defibrillation devices through skin incisions can produce high post-interventional pain levels [7,9]. Moreover, the number of such interventions is increasing [10]. Therefore, pain management has value for patients. In turn, patient welfare is crucial to hospital reputation and economic wellbeing. Pain management also has value for hospital staff.

We implemented a post-interventional pain management program in cardiac electrophysiology that achieved positive results; the proportion of patients who experienced moderate-to-severe pain decreased from 61% before the program to 47% after (*p* < 0.05) [8]. However, the barriers faced by project leaders, the group dynamics navigated, and steps along the road to the program’s implementation were not described. Therefore, the current paper aims to describe the process. Because pain-management program initiation represents a behavioral change intervention, and because such changes are difficult to accomplish, it is important to provide guidance to assist others in implementation of similar programs. We should note such programs can also be regarded as quality improvement.

Therefore, the objective of this paper was to analyze how the pain-management program was implemented in the electrophysiology clinic. The tools used derive from normalization process theory and social marketing. Normalization process theory, derived from sociology, aims to understand the mechanics by which complex interventions are implemented. The method explains how participants behave in the organizational and social context of those interventions. Social marketing aims to achieve the “greater good” by using marketing concepts to influence behavior. Such changes in practice are common in quality-improvement processes, and therefore our goal was to provide a template for others to implement similar change programs.

## 2. Methods

Because the methodology we employed will be unfamiliar to many healthcare professionals, we will deviate from the standard manuscript format. Instead, before describing the specific methods used, we will explain (1) the practice change setting, (2) why a practice change was needed, (3) outline the theory behind the approaches used to implement and assess practice change, and (4) introduce the language associated with normalization practice theory.

(1) *Practice change setting*. The Helios Heart Center Leipzig is a 440-bed tertiary referral center that specializes in general cardiology, electrophysiology, cardiac surgery, and pediatric cardiology. Our electrophysiology department treats approximately 3,000 patients per year. Most clinic patients suffer from cardiac arrhythmias that require interventional therapy; for example, ablation procedures for atrial fibrillation and ventricular tachycardia, and pacemaker and cardioverter defibrillator implantations.

The first author (KB) was assigned responsibility to obtain federal government certification for the electrophysiology department’s pain management program. The multidisciplinary team consisted of one physician from each hospital department, a nurse specialist in pain management, the head of nursing, the head of quality management, and an anesthesiologist. These personnel, together with the 45 nurses and 20 physicians who participated in the implementation of the program, were primary stakeholders. The hospital administration, although consulted throughout the process, was considered an indirect stakeholder.

(2) *Need for practice change*. An initial assessment of pain management practices was conducted in all departments by an external certification organization. This evaluation revealed quality targets for pain management had not been achieved. The initial reaction by senior physicians within the Electrophysiology Department was, “we do excellent work, and there is no need for pain management”. Physicians questioned the assessment methodology and disputed recommendations for a post-interventional pain-management program. In contrast, the department’s nurses stated that patients often reported moderate-to-severe pain after interventional procedures.

To address resistance among physicians, we conducted an additional survey, focused solely on patients’ needs. We expanded the sample size well above that of the original survey. This survey’s main finding was a high prevalence of post-procedural pain [7]. These new data served numerous purposes to illustrate the need for practice change. (1) Data forced physicians to appreciate there was indeed a problem with pain management that required attention. (2) Because the survey was conducted in-house, physicians considered the data legitimate. The results also validated the initial assessment. (3) The results enabled the consensus required to proceed with implementation of the program. (4) The data also revealed barriers to effective pain management from the perspectives of patients, nurses, and physicians. (5) The survey identified potential collaborative efforts that nurses, and physicians could adopt to benefit patients. The policy developed to initiate the program is outlined in reference [7].

(3) *Theory of the approaches used to implement change*. To embed complex interventions in complex settings requires collective, rather than individual, action [11]. Normalization process theory examines behaviors associated with new ways of implementation, conceptualization, or organization of practice. Such change includes collective action that results from complex patterns of social relations or interactions [12,13]. Application of normalization process theory can be assisted by social marketing tools. The latter are defined and categorized by the methodological program of the Cochrane Effective Practice and Organization of Care Review Group (EPOC) [14]. The framework provided enables comparison of research on behavioral change interventions.

(4) *Normalization Process Theory*. Normalization process theory represents a conceptual framework [15]. The intent is to promote understanding and explanations of dynamic processes involved in normalizing innovation [11]. The goal is to make complex interventions routine [13,16] and to embed them into social contexts [12]. The approach is content-specific, and thereby seeks to improve sustainability of change intervention [17] to the extent that the change becomes part of an organization’s culture [18]. Normalization process theory addresses the roles of individuals and groups; the realities of implementation of practice changes require both to succeed [19]. Consequently, the approach appeared well-suited to our quality improvement goal because it was used in previous successful programs [20].

There are four theoretical components to normalization process theory and each of the four components contains four elements. These 16 parts represent a considerable amount of information, which is most easily understood in practical terms (as described in Table 1). Therefore, here, we will outline them briefly:

(1) *Coherence/Sense Making*—represents how individuals and groups think, understand, and organize when implementing new processes; (a) *Differentiation*—understanding how the previous and proposed practices differ; (b) *Communal Specification*—involves the entire team working together to understand how new practices can be implemented; (c) *Individual Specification*—focuses on what is required of each person; (d) *Internalization*—appreciation of the value of the change.

(2) *Cognitive Participation*—the work involved to initiate the process, to organize and become involved, to contribute, and to stay involved to achieve the change. (a) *Initiation*—key participants should work to advance the project; (b) *Enrollment*—participants may need to be reorganized and appreciate how their work may change; (c) *Legitimation*—ensures that participants believe it is appropriate for them to be involved and that they can contribute; (d) *Activation*—participants must understand what is required to maintain practice change.

(3) *Collective Action*—how people interact and gain confidence with each other and with the elements of the change, and how resources and work are allocated. (a) *Interactional workability*—how participants interact with each other and the program; (b) *Relational integration*—how participants maintain trust in each other and in the program; (c) *Skill set workability*—the allocation of tasks is appropriate; (d) *Contextual integration*—ensures that allocation of resources is appropriate.

(4) *Reflexive monitoring*—appraisal of how the new program affects individuals and the organization. (a) *Systematization*—collection of information related to the program’s effects; (b) *Communal appraisal*—how participants evaluate data collected; (c) *Individual appraisal*—how participants evaluate program effects on them and their work; (d) *Reconfiguration*—does communal or individual appraisal result in attempts to change the program or practices within the program.

Each element can either assist or resist change because each element is derived from existing norms and conventions [12]. Knowledge of such norms and conventions help design interventions that are culturally acceptable to the group [23].

The steps, materials, and documentation described above were recorded at each stage in the process, which enabled precise recall [24]. This information was then coded according to the normalization process theory framework, adapted from earlier work [25,26,27,28]. The senior author (HA) conducted a cross-comparison [28] of the results to ensure their relation to the normalization theory framework. Similarly, the data were also coded according to the EPOC framework [14]. When that framework failed to reflect the tasks performed, we added additional marketing concepts. The collection and coding of the qualitative data addressed issues to do with validity, reliability and generalizability [29].

## 3. Results

Our analysis consisted of three facets. First, information on steps taken was coded and split into two sections. The first section divided processes involved in the pain-management program according to the 16 elements of normalization process theory described above: Table 1, column 3. The second section divided processes using the EPOC taxonomy and social marketing as they related to the normalization process theory elements (Table 1, column 4).

**Figure 1:**
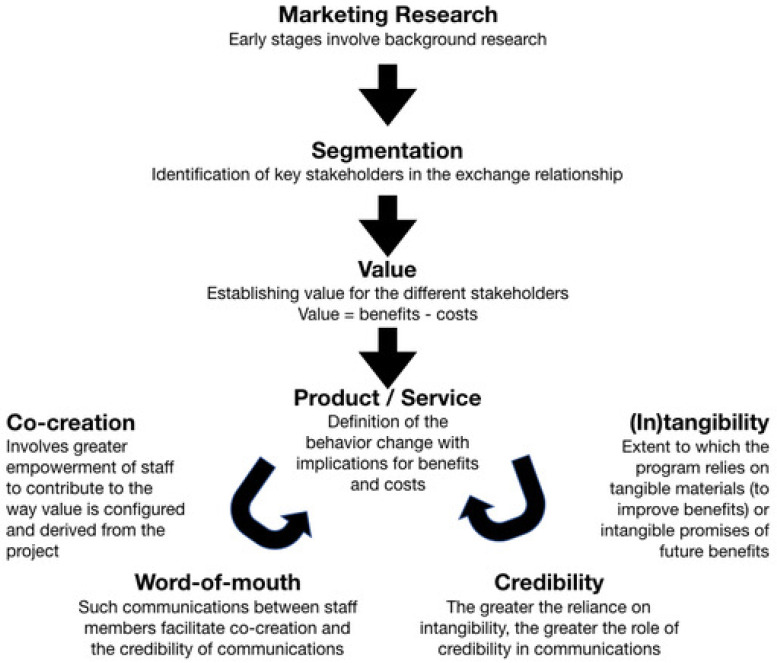
Social marketing concepts involved in the implementation of the behavior change intervention.

The third facet, performed in parallel with the second, focused on the social marketing. Figure 1 shows a schematic representation of our approach to marketing the program. The first step was research to identify the key stakeholders and establish their needs and wants. Subsequently, we determined the value each stakeholder group could derive from the program. In turn, this step enabled us to define the required behavior changes and their implications in terms of benefits and costs [30]. Two potential loops were identified. We initiated one of these loops to reinforce the changes and their value. We sought direct involvement of participants in as many aspects of the program as possible. In general, the greater the involvement, the greater the perceived value participants derived. The second loop involved provision of tangible benefits to stakeholders and promise of future benefits.

## 4. Discussion

Our analysis revealed how positive results from a quality-improvement project appeared consistent with successful application of normalization process theory. Furthermore, social marketing efforts enhanced the project. The program reduced the risk of late (between 8 and 24 h) post-interventional pain three-fold (odds ratio 0.32, 95% confidence intervals [0.16 to 0.64]; *p* = 0.001) [8].

Normalization process theory has typically been applied in the context of introduction of new interventions or practices [31]. However, the concept has also been extended to continuity of care [32]. Although such innovations can be regarded as quality improvement, direct connection between the two has seldom been made [33,34]. Since Donabedian’s seminal work [35], quality has been a focus of healthcare. Efforts to improve quality have likewise become a focus. Although methods for quality improvement, for example plan-do-study-act cycles, have been developed, the reasons behind the success or failure of such innovations remain obscure [36]. Many of the issues discussed in Siriwardena’s review [36] parallel the structure of normalization process theory. Similar parallels appear in a review of a proposed method of quality improvement in surgery [37]. Therefore, we speculated that the framework of normalization process theory could serve as a template for successful quality improvement. The advantage of normalization process theory, as emphasized by May, is not to provide a rigid set of instruction, but instead to offer a flexible framework [38].

Social marketing tools complement the use of normalization process theory. For example, our use of empirical data helped convince senior managers of the initiative’s value [39]. We were unable to offer monetary benefits. We instead emphasized the following: (1) adequate pain management is an ethical concern; (2) pain management is required for faster recovery; (3) patient satisfaction is crucial because it underpins future decisions on where to obtain healthcare [40]; (4) early pain assessment and treatment reduces patient dissatisfaction [8]; (5) patient dissatisfaction stresses hospital staff and thereby decreases job satisfaction.

In a previous study, nurses identified the inability to consult pharmacists on pain management as a barrier to effective therapy [41]. Likewise, Van Valen et al. revised their pain-management protocols such that nurses were not required to consult physicians [42]. Our protocols provided nurses with autonomy to administer pain medication, within defined limits, without physician approval. Nonetheless, physicians were tasked with ensuring adequate pain management. These combined efforts to address post-interventional pain underlined the abovementioned benefits; i.e., they serve to increase patient and staff satisfaction, and enhance hospital reputation [40,43].

Educational seminars helped develop proposals for standard operating procedures. Both nurses and physicians were able to incorporate ideas and suggestions as the program developed. This process enabled participants to derive value not only at the start of the program, but also as the program progressed [44], an illustration of value co-creation. Marketing concepts also played a role in the seminars; for example, exchange and “get” notions [45,46]. In these interactions, value is defined as the benefit received minus the cost incurred. For patients, the benefit is less pain minus the cost of providing personal information to nurses. For nurses, the benefit is increased patient satisfaction and the cost is time spent asking patients about pain and chart documentation [47,48,49]. Because we demonstrated significant decreases in the post-interventional pain after successful implementation of our pain-management program, it is reasonable to assume all stakeholders would perceive the change as beneficial.

Analysis of behavior change interventions with normalization process theory and the EPOC approaches has been reported at population [50] and organization [51] levels. These sociology-based theories help understand the implementation, integration, and consolidation of innovation in healthcare [52]. We applied the theories to behavior change intervention to reveal the dynamics and barriers to, and facilitators of, change. We employed a participatory approach. Participatory normalization process theory methods were recently used to incorporate an electronic patient-reported outcome measure in routine care [53].

In the step-coding process, we did not assign processes to every normalization process theory category—an issue described by other investigators [54]. Although such omissions could be perceived as weakness, one strength of normalization process theory is that it permits coding flexibility, and analysis should consider context [20]. Furthermore, because implementation processes are dynamic, the expectation that a single theory covers all aspects of all processes is unrealistic.

In this context, we should acknowledge that normalization process theory can be regarded as one element of the broader field of implementation science [55]. Implementation science’s goal of translating clinical innovation into clinical practice matches what we sought to achieve. There are multiple strategies [56], frameworks, and theories [57] that can be applied in implementation science. The appropriate choices will likely be determined by context.

Marketing: Our analysis added two concepts to the EPOC framework to explain the efficacy of marketing tools. Tangibility: the participants’ ability to define and describe the program and process will aid their understanding of its potential attributes and value [58,59]. Our use of seminars and distribution of educational material contributed to tangibility. Of course, tangibility cannot always be achieved and sometimes only intangibles can be offered, such as promises of future benefits [60]. However, for the latter to be effective, the change agent must be trusted and credible, as shown by the second novel concept in Figure 1.

Persistent Challenges: We, like many academic medical facilities, face the challenge of considerable staff turnover. Therefore, to maintain adherence requires constant effort. Current and new staff require frequent training sessions and motivation to continue with the development of normalization process theory. Nonetheless, despite these persistent challenges, diligent application of our implementation strategy resulted in persistence of quality improvement in the pain management program, as we described [8].

## 5. Conclusions

Implementation of change and quality-improvement initiatives invariably present challenges. We demonstrated successful intervention to change behavior, and improve quality, based upon normalization process theory and adaptation of tools from social marketing and from the EPOC. We propose that concepts from marketing disciplines will provide further insights into how acceptability of such behavioral change and quality improvement interventions can be enhanced. Specifically, concepts of tangibility and co-creation enable change instigators to focus on the clarity of the processes and outcomes of change and thereby explain the relevance to stakeholders.

## Figures and Tables

**Table 1:** NPT elements relevant to the program, and the EPOC and social marketing tools used.

NPT Group	NPT Element	Relevance to This Project	EPOC and Social Marketing Tools
**Coherence/Sense-making**	**Differentiation** (*Participants distinguish the intervention from current way of working.*)	Initially, senior physicians (opinion leaders) questioned the results of the certification organization’s initial assessment and the need for a pain management program. To overcome this initial resistance, the project leader carried out a survey to investigate patients’ needs. This survey indicated a high prevalence of post-operative pain.	**(PI) Marketing (Research)** We adapted the EPOC term “marketing” and considered “marketing research” as the process of determining the needs and wants of different stakeholders. The revised term emphasizes the importance of this preliminary stage.
		After implementation of this initiative, nurses and physicians had a structured approach to pain management. Before, pain management did not play a crucial role and depended on provider knowledge and enthusiasm for pain management, and patients’ request for pain medication.	**(POI) Presence and functioning of adequate mechanisms for dealing with patients’ suggestions and complaints** The survey of patient opinion helped justify the intervention.
	**Communal specification** (*Participants collectively agree on the purpose of the intervention*)	We presented the concept of PMP (needed because of poor results from a pre-certification survey) at noon rounds, during which junior and senior doctors meet to discuss patient cases and clinic operation.	**(PI) Local opinion leaders (*Providers nominated by their peers as “educationally influential”*)** The opinions of senior staff carried legitimacy and credibility, and therefore would be accepted.
	**Individual specification** (*Participants understand what the intervention requires of them.*)	Every physician received a red cardboard-letter (to attract attention) with take-home messages and KB’s telephone number in case of questions. Doctors were also given a pocket card with the pain assessment scale on one side and a list of World Health Organization painkillers adopted by the hospital on the other side.	**(PI) Reminders** (*Patient or provider encounters provided specific information designed, or intended, to prompt a health professional to recall information, or perform, or avoid an action to aid patient care.*) The reminders were used to aid recall of actions related to the intervention.
		Doctors and nurses were educated separately to address their different needs and expectations.	**Segmentation** [21] This standard marketing concept is not in the EPOC list. It refers to the recognition of differences in the needs of different stakeholders.
		Educational materials (Power Point presentations) were delivered to every staff member via email after the educational seminars.	**(PI) Distribution of educational material** This was effective given the required communication that had to be addressed.
	**Internalization** (*Participants assign value to the intervention for their work*.)	Because the program makes life easier for doctors and nurses there are no specific resources necessary. Uncoordinated actions before implementation of the program provoked delays in pain level assessment and administration of pain medication. These delays could be minimized by structured pain-level assessment and giving prescription medications the evening before the intervention.	**(SI) Changes in scope and nature of benefits and services.** The benefits stakeholders derived from behavior change had to be recognized to demonstrate their value. Recognition is vital because of the costs (time needed for pain assessment and medication prescription) associated with the intervention.
**Cognitive participation**	**Initiation** (*Key individuals drive the intervention forward.*)	A pain nurse from the Anesthesiology Department was in regular contact with the director of the PMP, an anesthesiologist. Their aim was to establish better practice in the Cardiac Surgery Department first, and then the entire Heart Center. The Heart Center’s management wanted to achieve certification as a qualified pain management facility. Therefore, a task force was set up; composed of a pain nurse, the anesthesiologist, one doctor from every department, the head of quality management, and the head of nursing. They organized meetings, and managed the development of SOP.	**(OI) Clinical multidisciplinary teams** (*Creation of a new team of health professionals from different disciplines, or addition of new members to the team. Team members work together to care for patients.*)
	**Enrolment** (*Participants agree that the intervention should be part of their work.*)	Senior management created the pain nurse position. This position did not exist prior to the intervention.	**(PI) Local opinion leaders + (OI) Skill mix changes** Buy-in from opinion leaders enabled provision of a useful resource, specifically the addition of skillsets available for the project.
	**Legitimation** (*Participants buy into the intervention, and believe it is right for them to be involved and that they can make a valid contribution.*)	This was the first time the Heart Center applied for certification, which was received ten months after the first assessment. The certifying organization had approved other hospitals for pain management, and they provided a basis for comparison. The organization is known in its field, but unknown to hospital staff except for the anesthesiologist and the pain nurse. As the project progressed, staff became more familiar with the organization. Hospital staff did not question the organization’s background.	**(SI) Presence and organization of quality monitoring mechanisms** Knowledge of the certification process motivated the staff to engage in the project.
		KB presented suggestions about pain management to the staff and invited them to develop these ideas. This feedback was used to adapt our program.	**(PI) Local consensus process** Participants were included in discussions to ensure they agreed the problem was important, and that the management approach was appropriate. Their contributions, both prior to and during the program, represented “co-creation”.
**Collective action**	**Interactional workability** (*Participants perform the tasks required by the intervention*.)	Nurses and doctors assessed patients’ charts every evening and were able to add missing information on medication prescriptions. If necessary, nurses could remind physicians.	**(SI) Staff organization** **(OI) Formal integration of services** (*Bringing together services across sectors or teams, or the unification of services; this is sometimes called “seamless care”.*) Intervention processes adopted resulted in more seamless care.
	**Relational integration** (*Participants maintain trust in each other’s work and expertise throughout the intervention.*)	Nurses and doctors work in a complex environment. They are confronted with significant administrative duties, and participate in quality-improvement measures. Pain management requires teamwork. We argued that asking brief questions about patients’ pain costs only seconds. In contrast, the gain is relatively large and should improve treatment quality and prevent further pain.	**Establishment of value for the different stakeholders** Asking brief questions helped reduce a perceived cost (time) and thus improved value for one group of stakeholders.
	**Skillset workability** (*The tasks of the intervention are appropriately allocated to participants.*)	Clinicians were advised to (1) pay close attention to continuation of current pain medication, (2) prescribe complete recommended doses of pain medication, (3) inform patients on all administered pain medications.	**(PI) Educational seminars and SOP; distribution of educational material.** Helps improve the tangibility of the change required.
		Nurses were advised to ask patients about pain intensity after interventions by using a numeric scale every two hours on the day of procedure (they already checked wounds and foot pulses). Nurses also evaluated and documented pain intensity at least every 12 h.	**(PI) Patient-mediated intervention** (*New information, not previously available, collected directly from patients and given to the provider.*)
	**Contextual integration** (*The intervention receives adequate organizational support.*)	Audits were performed annually (two internal, one external) to assess the adoption of the policy, provide feedback, and discuss emerging problems with staff.	**(PI) Audit and feedback** (*Any summary of clinical performance of health care over a specified period.*)
**Reflexive monitoring**	**Systemization** (*Participants receive data on the intervention’s effects.*)	The hospital achieved certification (external audit) on structured pain management complying with the requirements of the certification organization.	**(SI) Ownership, accreditation, and affiliation status of hospitals and other facilities**
	**Communal appraisal** (*Participants collectively assess the intervention as worthwhile*.)	Results of the last certification were presented as a collective achievement. People can see the certificates; they were posted in the hospital.	**(POI) Provider satisfaction of work conditions and the material and mental rewards** (*e.g., interventions to boost morale*)
**Other**		Patients received structured information in the form of educational material. In addition, oral explanations were provided by nurses and doctors. These included descriptions of the pain measurements and treatment methods. The staff also emphasized that patients should disclose any pain they experienced.	**Mass media in the form of leaflets; marketing via word of mouth with linear marketer influence model** [22]**; oral reminders.**

Specific terms are given in bold, and explanations are presented in italics if necessary. OI, organizational intervention; PI, professional intervention; POI, patient-oriented intervention; SI, structural intervention; PMP, pain management program; SOP, standard operating procedures.

## Data Availability

All data generated or analyzed during this study are included in this published article.
